# Practical and statistical aspects of subgroup analyses in surgical neuro-oncology: A comprehensive review from the PIONEER consortium

**DOI:** 10.1093/neuonc/noae261

**Published:** 2024-12-07

**Authors:** Jasper K W Gerritsen, Philipp Karschnia, Jacob S Young, Martin J van den Bent, Susan M Chang, Timothy R Smith, Brian V Nahed, Jordina Rincon-Torroella, Chetan Bettegowda, Nader Sanai, Sandro M Krieg, Takashi Maruyama, Philippe Schucht, Marike L D Broekman, Joerg-Christian Tonn, Patrick Y Wen, Steven De Vleeschouwer, Arnaud J P E Vincent, Shawn Hervey-Jumper, Mitchel S Berger, Rania A Mekary, Annette M Molinaro

**Affiliations:** Department of Neurosurgery, Erasmus Medical Center, Rotterdam, The Netherlands; Department of Neurosurgery, University of California, San Francisco, California, USA; Department of Neurosurgery, Ludwig-Maximilian University Hospital, Munich, Germany; Department of Neurosurgery, University Hospital Erlangen, Erlangen, Germany; Department of Neurosurgery, University of California, San Francisco, California, USA; Department of Neurology, Erasmus MC Cancer Institute, Rotterdam, The Netherlands; Department of Neurosurgery, University of California, San Francisco, California, USA; Department of Neurosurgery, Brigham and Women’s Hospital, Boston, Massachusetts, USA; Department of Neurosurgery, Massachusetts General Hospital, Boston, Massachusetts, USA; Department of Neurosurgery, Johns Hopkins University, Baltimore, Maryland, USA; Department of Neurosurgery, Johns Hopkins University, Baltimore, Maryland, USA; Department of Neurosurgery, Barrow Neurological Institute, Phoenix, Arizona, USA; Department of Neurosurgery, University Hospital Heidelberg, Heidelberg, Germany; Department of Neurosurgery, Tokyo Women’s Medical University, Tokyo, Japan (T.M.); Department of Neurosurgery, Inselspital University Hospital Bern, Bern, Switzerland; Department of Neurosurgery, Haaglanden Medical Center, The Hague, The Netherlands; Department of Neurosurgery, Leiden University Medical Center, Leiden, The Netherlands; Department of Cell and Chemical Biology, Leiden University Medical Center, Leiden, The Netherlands; Department of Neurosurgery, Ludwig-Maximilian University Hospital, Munich, Germany; Department of Neuro-Oncology, Dana-Farber Cancer Institute, Boston, Maryland, USA; Department of Neurosurgery, University Hospitals Leuven, Leuven, Belgium; Department of Neurosurgery, Erasmus Medical Center, Rotterdam, The Netherlands; Department of Neurosurgery, University of California, San Francisco, California, USA; Department of Neurosurgery, University of California, San Francisco, California, USA; Department of Neurosurgery, Brigham and Women’s Hospital, Boston, Massachusetts, USA; Department of Pharmaceutical Business and Administrative Sciences, School of Pharmacy, MCPHS University, Boston, Maryland, USA; Department of Epidemiology and Biostatistics, University of California, San Francisco, California, USA

**Keywords:** confounding, malignant glioma, multiplicity, study design, subgroup analysis

## Abstract

Subgroup analyses are essential to generate new hypotheses or to estimate treatment effects in clinically meaningful subgroups of patients. They play an important role in taking the next step toward personalized surgical treatment for brain tumor patients. However, subgroup analyses must be used with consideration and care because they have significant potential risks. Although some recommendations are available on the pearls and pitfalls of these analyses, a comprehensive guide is lacking, especially one focused on surgical neuro-oncology patients. This paper, therefore, reviews and summarizes for the first time comprehensively the practical and statistical considerations that are critical to this field. First, we evaluate the considerations when choosing a study design for surgical neuro-oncology studies and examine those unique to this field. Second, we give an overview of the relevant aspects to interpret subgroup analyses adequately. Third, we discuss the practical and statistical elements necessary to appropriately design and use subgroup analyses. The paper aims to provide an in-depth and complete guide to better understand risk modeling and assist the reader with practical examples of designing, using, and interpreting subgroup analyses.

Neurosurgeons can choose from an array of surgical modalities and techniques to achieve maximal safe resection of brain tumors. For example, techniques can be used to improve the extent of resection (eg, intraoperative MRI,^1,2^ intraoperative fluorescence,^3^ Raman spectroscopy,^4,5^ or intraoperative ultrasound^6,7^), to prevent neurological deficits (eg, evoked potentials,^8–10^ minimally invasive techniques such as Laser Interstitial Thermal Therapy [LITT]^11,12^), or both (awake or asleep brain mapping^13,14^). There is increasing evidence on the role of certain aspects of surgery for neuro-oncological patients, such as the extent of resection, supramaximal resection, and intraoperative mapping.^15–21^ At the same time, there have been notable improvements in classifying brain tumor patients based on molecular information.^22–24^ These developments warrant new, comparative analyses of different surgical treatments in these newly classified patient subgroups.

Traditionally, studies have evaluated the benefits of these surgical treatments in overall cohorts of brain tumor patients.^1,3,6^ Although this is a powerful approach to demonstrate the overall benefit of a treatment, it fails to inform the neurosurgeon for which individual patient it would be beneficial, and for which it is unlikely to improve outcome.^25,26^ This means that the specific effects of these treatments in important subgroups of patients—for example, based on age, preoperative functioning status, or molecular mutation profile—remain unclear. This, in turn, might lead to the underuse of these modalities due to uncertainty about their benefits, even though these approaches could potentially improve the outcomes of selected patients. For example, recent evidence shows that maximal and supramaximal may be more important for astrocytoma grade 2 patients than oligodendroglioma grade 2 patients.^20^

On the other hand, this might also lead to the overuse of invasive modalities in patients who might have benefitted from avoiding invasive procedures. This might be illustrated by a recent study demonstrating that only patients <65 years old benefitted from supramaximal resection.^16^ Subgroup analyses can also provide insight into treatment effects in specific patient populations, informing risk stratification for prospective trials. For example, a clinical risk score for postoperative outcomes in glioblastoma patients can decrease prognostic imbalance between study arms.^15,26^ Last, post-hoc exploratory subgroup analyses can generate new hypotheses that can be validated in subsequent confirmatory studies.^27^

It is essential to carefully design and interpret these subgroup analyses, as they are often exploratory post-hoc analyses with inherent statistical risks.^28–30^ This highlights the need for a solid understanding of these analyses to ensure adequate design and interpretation. While certain aspects of subgroup analyses have been previously discussed in the literature, a comprehensive guide with practical pearls and pitfalls—especially one focused on surgical neuro-oncology patients—remains lacking. The fragmentation of existing recommendations can lead to improper use, selective reporting, and misinterpretation, which may harm clinical practice. This review aims to fill that gap by providing, for the first time, a comprehensive guide on planning, analyzing, and interpreting subgroup analyses in the context of surgical neuro-oncology studies. This guide has been put together by the members of the international PIONEER Consortium (Personalized Interventions and Outcomes in Neurosurgical Oncology Research Consortium), an updated name of the previously known ENCRAM Consortium (European and North American Consortium for Intraoperative Mapping in Glioma Patients) that is more representative of the expanded and changing scope of the Consortium. For this manuscript, we focus on subgroup analyses as stratified analyses of a study population of interest. We also evaluate various study designs and types of subgroup analyses, highlighting their advantages, challenges, pearls, and pitfalls in a practical setting.

## Advantages and Challenges of Different Study Designs in Surgical Neuro-

Randomized controlled trials (RCTs) are challenging for any surgical intervention, and this also holds true for neuro-oncology. The need for individual and clinical equipoise greatly limits the options for control groups and blinded randomization is nearly impossible.^31^ Individual equipoise means that the treating physician is truly uncertain about which treatment might be the best for a specific patient. In contrast, clinical equipoise embodies a similar uncertainty but expanded to the profession.^31–33^ As such, only a handful of RCTs have been published evaluating surgical modalities’ benefit in glioblastoma patients.^1,3,34^ There are a few actively enrolling RTCs for glioblastoma patients: for instance, the SAFE trial is investigating the potential benefits of awake craniotomy (NCT03861299),^35^ the RESURGE trial is investigating re-resection in recurrent tumors (NCT02394626), and the BOLD and G-SUMIT trials are comparing supramaximal resection to other resections (NCT04243005 and NCT04737577, respectively). Table 1 summarizes key RCTs’ design and study protocol and prospective cohort studies on resection for newly diagnosed diffuse gliomas.

**Table 1.** Design and Protocol of Prospective Key Studies on Resection for Newly Diagnosed Gliomas

Study	Study design	Key intervention analyzed	Key surgical findings	Relevant subgroups analyzed	Pre-specified statistics
Glioblastoma					
NCT02379572 Roder et al.^2^	Non-randomized, parallel cohort controlled trial (glioblastoma: *n* = 277)	ioMRI-guided resection vs. 5-ALA-guided resection	No difference in complete resection rate (81% vs. 78%; OR 1·09, CI: 0·57–2·08; *P* = ·79) or OS (HR 1·00, CI: 0·64–1·55; *P* = ·99) between ioMRI vs. 5-ALA	OS and PFS stratified for different amount of residual CE tumor volume OS stratified for residual tumor volumes in subgroups defined by MGMT promotor status	Applied statistical tests pre-defined Sample size calculation a priori done No pre-defined protocol for subgroup analysis
RESECT (NCT01811121) Picart et al.^36^	Randomized, single-blinded, controlled phase III trial (glioblastoma: *n* = 171)	5-ALA-guided vs. conventional white-light guided resection	Higher rate of complete resection of the contrast enhancement using 5-ALA: 79% vs. 48% (absolute difference 29%, CI: 17–40; *P* < 0·0001). Complete resection was associated with higher OS (HR 0·65, 0·42–1·01; *P *= ·05)	No subgroup analysis done	Unclear whether statistical tests pre-defined Sample size calculation a priori done No pre-defined protocol for subgroup analysis
GGN (no NCT available) Kreth et al.^37^	Prospective longitudinal cohort study (glioblastoma: *n* = 273)	Complete vs. subtotal resection vs. biopsy	Complete resection was associated with higher median OS: 17·1, CI: 12·6–21·5 vs. 11·7, CI: 10·0–13·5 months (*P* = ·001) No differences in OS between subtotal resection and biopsy (*P* = ·1)	Outcome stratified for extent of resection in following subgroups: Treatment regimens MGMT promotor status	No pre-defined statistical protocol No sample size calculation No pre-defined protocol for subgroup analysis
NCT01394692 Senft et al.^38^	Randomized, open-label, controlled trial (glioblastoma: *n* = 46; anaplastic astrocytoma: *n* = 1, anaplastic oligodendroglioma: *n* = 1)	ioMRI-guided vs. conventional white-light guided resection	Complete resection more frequent with ioMRI guided resection as compared to conventional resection: 96% vs. 68%, *P* = ·023 Higher 6-month PFS with ioMRI guided resection as compared to conventional resection (67% vs. 34%; OR 0·28, CI: 0·09–0·91; *P* = ·046)	Rate of complete resections compared in junior vs. senior neurosurgeons Rate of residual tumor depending on the use of neuronavigation Outcome stratified for extent of resection in the overall cohort and in newly diagnosed grade IV tumors only	Unclear whether statistical tests pre-defined Sample size calculation a priori done No pre-defined protocol for subgroup analysis
NCT00241670 Stummer et al.^39^	Post hoc analysis of the trial by Stummer et al. (glioblastoma: *n* = 243 from per-protocol cohort)	Complete vs. subtotal resection	Complete resection was associated with higher median OS: 16·7, CI: 13·4–19·0 vs. 11.8, CI: 10·4–13·7 months (*P* < .0001; HR 1·75, CI: 1·26–2·44; *P* = ·0004)	Outcome stratified for extent of resection in the following subgroups: 5-ALA (vs. white-light) Eloquent (vs. non-eloquent) > 60 years of age (vs. < 60 years)	No pre-defined statistical protocol for post hoc analysis Post hoc analysis: rested upon available cohort from NCT00241670 No pre-defined protocol for post hoc subgroup analysis
NCT00241670 Pichlmeier et al.^40^	Post hoc analysis of the trial by Stummer et al. (glioblastoma: *n* = 243 from per-protocol cohort)	Complete vs. subtotal resection	Overall cohort: complete resection was associated with higher median OS: 16·7, CI: 14·3–19·0 vs. 11·8, CI: 10·4–13·7 months (*P* < ·0001) Subgroup analysis: complete resection associated with higher median OS in the RTOG-RPA class IV and V (IV: 17·7, CI: 14·3–22·5 vs. 12·9, CI: 10·3–14·7; V: 13·7; CI: 8·3–17·6 vs. 10·4, CI: 8·1–11·; *P* = ·0007).	Outcome stratified for extent of resection in the subgroups determined per RTOG-RPA (class III-V)	No pre-defined statistical protocol for post hoc analysis Post hoc analysis: rested upon available cohort from NCT00241670 No pre-defined protocol for post hoc subgroup analysis
NCT00241670 Stummer et al.^3^	Randomized, controlled trial (grade III/IV: *n* = 270 in full-analysis cohort, including *n* = 137 glioblastomas)	5-ALA-guided vs. conventional white-light guided resection	Higher rate of complete resection of the contrast enhancement in the 5-ALA arm: 65% vs. 36% (absolute difference 29%, CI: 17–40, *P* < ·0001) 6-months PFS higher in 5-ALA arm: 41·0%, CI: 32·8–49·2 vs. 21.1%, CI: 14·0–28·2 with an absolute difference of 19·9%, CI: 9·1–30·7; *P* = ·0003	Outcome as well as frequency of and time to re-resection stratified for type of surgery in the following subgroups: Eloquent (vs. non-eloquent) > 55 years of age (vs. < 50 years) KPS > 80 (vs. 70–80) Outcome stratified per residual tumor volume	Applied statistical tests pre-defined Sample size calculation a priori done Subgroup analyses based on factors used for randomization
Astrocytoma and oligodendroglioma					
Shaw et al.^41^	Prospective longitudinal cohort study (astrocytoma grade II: *n *= 61, oligodendroglioma grade 2: *n *= 50)	Complete vs. subtotal resection	Residual tumor volume ≥ 1 cm predictive for PFS (HR 3·54, CI: 1·83–6·84; *P* = ·0002)	Outcome stratified per residual tumor volumes in the following subgroups: Astrocytomas Oligoastrocytomas Oligodendrogliomas	Unclear whether statistical tests pre-defined No sample size calculation Unclear whether pre-defined protocol for subgroup analysis

Abbreviations: 5-ALA = 5-aminolevulinic acid; CE = contrast enhancing; CI = confidence interval; EOR = extent of resection; FLAIR = fluid-attenuated inversion recovery; HR = hazard ratio; ioMRI = intraoperative magnetic resonance imaging; KPS = Karnofsky Performance Score; MGMT = 06-methylguanine-DNA-methyltransferase; NCE = non-contrast enhancing; OR = odds ratio; OS = overall survival; *P* = *P*-value; PFS = progression-free survival; RPA = recursive partitioning analysis; RTOG = Radiation Therapy Oncology Group; SD = standard deviation; STR = subtotal resection; WHO = world health organization.

Pubmed was searched for prospective cohorts of patients with newly diagnosed glioblastoma, astrocytoma grade 2–4, or oligodendroglioma grade 2–3. Only papers published after 2005 (following the introduction of the EORTC 26,981/22981-protocol for concomitant radiochemotherapy in glioblastoma) with data on extent of resection were included. Database closure was January 1, 2024. Study names, design, key intervention, key findings, relevant subgroups analyzed, and pre-specified statistics are indicated. 95%-confidence intervals as measures of uncertainty are given within “key results”.

**Figure 1.**
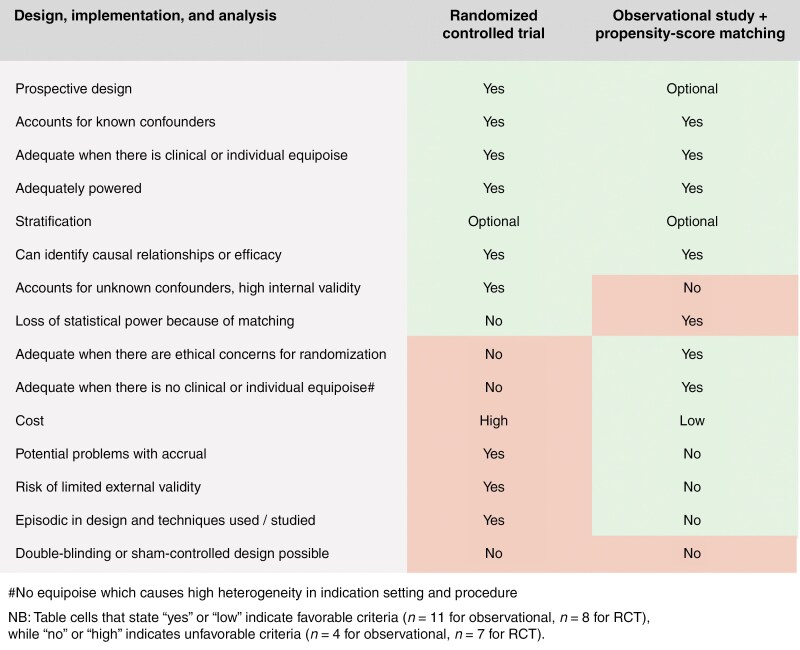
Comparison of study designs in surgical neuro-oncology.

It is undisputed that RCTs have major strengths: it cannot be overstated that they are the only study design that can account for both known and unknown confounders due to randomization, unlike observational studies that can account for only known confounders. Additionally, they can be combined with blinding techniques (single-blind, double-blind, and triple-blind), although complete blinding is not feasible for surgical studies in practice.^42^ When preceded by appropriate power analyses, they are the best approach for comparing the overall benefit of different treatment arms with high internal validity.^43^ However, several issues make designing and completing RCTs for investigating surgical neuro-oncological modalities particularly difficult.^44^ Critically, the lack (or perceived lack) of equipoise often results in highly selective study recruitment, slow accrual and lack of external generalizability.^45–48^ Moreover, many RCTs suffer from slow accrual due to the highly selective inclusion of patients with exceptional high-performance statuses. As a result, a considerable proportion of RCTs fail to be completed across neuro-oncology.^49,50^ The effect of a lack of patient acceptance to be randomized in the context of brain tumor surgery is another barrier. Critically, RCTs are extremely costly and require significant resources and time to complete relative to observational studies with carefully selected propensity-matched groups.^51^ Finally, RCTs cannot include updates on the techniques when the trial is active (also called “episodic design”), which can limit the inclusion of new technology or fail to account for a change in equipoise during trial enrollment, potentially making any results outdated before they are published. Given these limitations, RCTs may be best applied to questions for very specific subgroups of patients in which there is a true equipoise of treatment, where broad generalizability is not necessary, and a large multicenter effort can accelerate patient recruitment. Randomized controlled trials can be followed by post-hoc exploratory, hypothesis-generating subgroup analyses and subsequently, large observational studies with pre-defined subgroups to assess the risk-benefit ratio for selected patient subgroups. When there is a lack of equipoise and a large heterogeneity in clinical practice, prospective observational studies coupled with matching methods might be better suited to answer the research question. Notably, it has been shown that well-designed observational studies may find a similar magnitude of the associations between exposure and outcome as RCTs.^52,53^Figure 1 evaluates the methodological strengths and weaknesses of randomized versus observational study designs in surgical neuro-oncology.

## Reasons to Use Subgroup Analyses

Generally, there are 4 main reasons for subgroup analyses, as described comprehensively by Rothwell in 2005.^54^ The first reason is possible heterogeneity in treatment effects related to risk. This poses an additional challenge because study arms are often skewed in the risk distribution within the study arms. This means that a disproportionate (low) number of patients is responsible for a disproportionate (high) risk. As a consequence, the treatment effect might be over-interpreted for low-risk patients.^55^ The long-standing debate on biopsy versus resection for elderly glioblastoma patients could be an example.^56,57^ Within this subgroup, the location of the tumor causes possible heterogeneity in treatment effect: deep-seated, butterfly, or basal ganglia tumors have vastly different risk profiles than cortical tumors. This difference in risk warrants an additional subgroup analysis to adequately compare resection versus biopsy. The second reason is closely related: instead of differences in risk, there are potential differences in pathophysiology among the patients. Our previous publications might be practical examples.^14,19^ We demonstrated that awake mapping and maximal resection only led to longer survival outcomes in patients with 06-methylguanine-DNA-methyltransferase (MGMT) methylated tumors, but not in MGMT unmethylated tumors. We hypothesized that the cytoreduction synergized with the adjuvant treatment only in MGMT-methylated patients.

The third reason is a clinically important question regarding the practical application of the treatment, such as differences in risk-benefit ratios across patients due to age categories or surgery timing. For instance, Molinaro and coauthors found that supramaximal resection conferred a survival advantage only among patients 65 years or younger.^16^ Young and coauthors found that a longer time between diagnosis and surgery did not negatively impact survival in glioblastoma patients.^58^ Importantly, they concluded that “future studies are needed to explore subgroups for whom time-to-surgery may impact clinical outcomes.” The fourth reason consists of the underuse of the treatment in specific groups. In neurosurgical oncology, for example, tumors presumed inoperable by some surgeons or centers may benefit from surgical resection. Krieg et al. showed that a safe resection was possible in most of these patients in expert centers.^59^ Additionally, Southwell et al. showed that the use of intraoperative mapping could even lead to not only a safe but also a maximum resection in a high percentage of these patients.^60^

**Figure 2.**
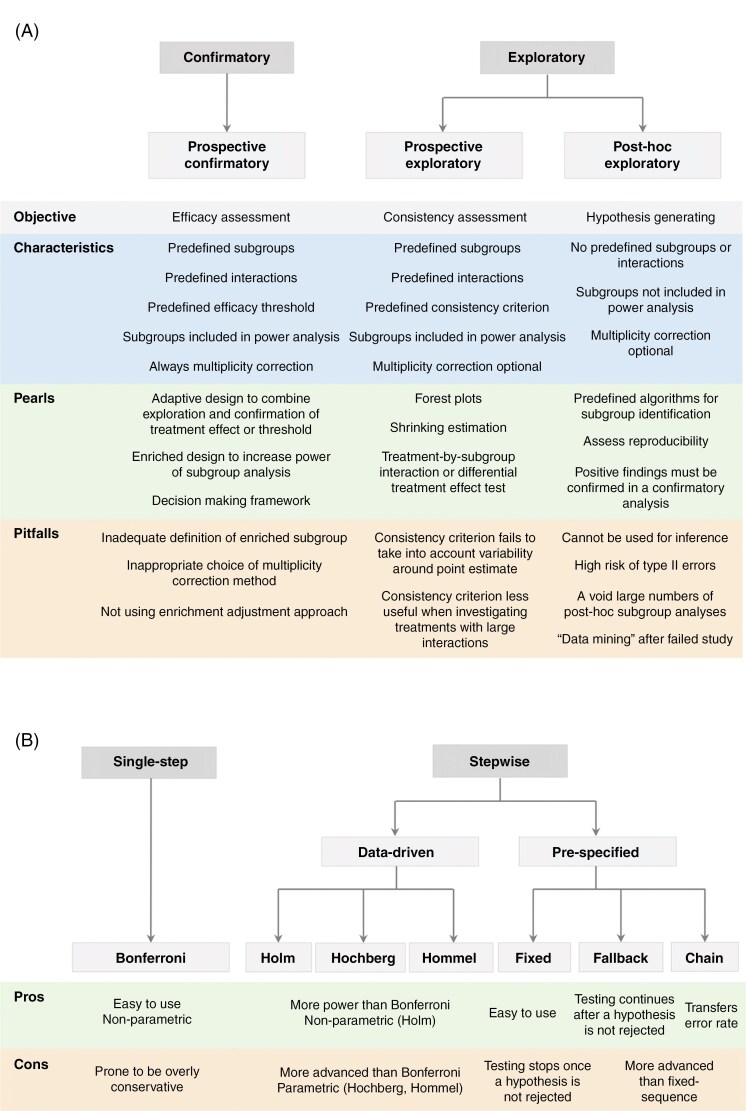
(A) Subgroup analysis methods. (B) Multiplicity correction methods.

The following paragraphs will elaborate on 3 methods of subgroup analysis: (1) prospective and confirmatory subgroup analyses (inferential subgroup analysis), (2) prospective and exploratory subgroup analyses (consistency assessment and supportive subgroup analysis), and (3) post-hoc, exploratory subgroup analyses.^61,62^ These 3 types of subgroup analysis^61^ (Figure 2A) are powerful methods that can be employed for both randomized and observational study designs but need to be designed, performed, and interpreted cautiously.

## The 3 Types of Subgroup Analysis

### (1) Prospective, Confirmatory Subgroup Analyses

With prospective subgroup analyses, the researchers define the subgroups they are interested in and their expected direction of treatment effect before the study starts (also called pre-defined subgroups). Often, these subgroups are defined by systematically evaluating previous evidence and identifying all potentially relevant factors (in a clinical or biological sense) and their potential interactions. The primary aim of the prospective, confirmatory subgroup analysis is to determine the efficacy of a certain treatment, surgery, or other intervention within a specific patient population. This requires comprehensive methodological planning and correcting for multiple tests to study causal relationships (inferential analyses).

There are 2 general risks when planning prospective subgroup analyses: type I and type II errors. Type I errors (false positives) are often caused by studying too many different subgroups and carry the risk of overinterpretation of the results.^29,63,64^ Type II errors (false negatives) are frequently the consequence of inadequate power because the subgroups were not considered during the sample size calculation. This leads to underpowered analyses and unreliable results.^29^ This is an inherent weakness with post-hoc subgroup analyses (elaborated upon later); however, prospective studies (discussed here) can pre-empt this problem by considering the subgroups when planning the study’s sample size.

Trial design is one of the key considerations when planning a prospective subgroup analysis. In a fixed design trial, the study design is documented before the start of the study and no modifications are allowed during the trial. Often, these trials only include the specific subgroup that the researcher is interested in, for example, to study the benefit of resection in elderly patients >80 years of age (single population).^57^ On the other hand, studies can include multiple subgroups simultaneously (multi-population). For example, the currently accruing SUPRAMAX study investigates the benefit of supramaximal resection in 3 pre-defined subgroups: age, MGMT methylation status, and preoperative Karnofsky Performance Score (KPS) status.^65^

The alternative to a fixed design is an adaptive trial design. This means that the study design can be modified during the study by using an interim analysis at a time that was decided before the start of the trial.^66,67^ It is up to the statistical and clinical committees to decide about the timing of the interim analysis. This is done in order to detect an early trend in the data to decide whether the trial needs to be stopped or modified. The timing of this varies by the research question, study team/statistician, the specific design, the expected recruitment and event rates, the length of time to assess the outcome, and other ethical considerations, without inflating the type I error rate or compromising power.^68^ Notably, if the analysis is conducted too early, it may lack sufficient power to detect any statistical significance and may erroneously lead to premature stopping. Hence, controlling for increased type I and type II errors while calculating sample size is needed.^69,70^ An example is adaptive randomization, in which the group allocation is altered based on the interim analysis and more (or even all) patients are allocated to the treatment group that is performing better (“drop-the-loser”).^71^ To our knowledge, neurosurgical trials have yet to use an adaptive design, but these are already utilized in neuro-oncological trials. For example, Rahman et al. used adaptive randomization in their study to compare abemaciclib, neratinib, and CC-115 (with chemotherapy plus radiotherapy as control arm) for their efficacy as adjuvant treatment in newly diagnosed glioblastoma patients.^72^ They started the trial with a 1:1:1:1 randomization allocation, which was then automatically adapted during the trial. Based on the interim results, CC-115 had inferior progression-free survival (PFS) than the other treatment arms. Consequently, the randomization probability for this treatment arm decreased from 25% to 16% and as a result, 12 instead of 18 patients were included in the CC-115 arm.

Adaptive trial designs can be advantageous for subgroup analyses in neurosurgical studies.^71^ For example, they can reduce the required sample size with the same power by a drop-the-loser design or re-estimating the sample size based on the interim analysis. Decreasing or eliminating randomization to inferior treatment arms also helps accrual and acceptance of patients to be randomized, which are notable issues for neurosurgical trials.^71^ A second advantage is the possibility to combine in one study both exploration and confirmation of (1) a treatment effect (eg, effect of resection versus biopsy in thalamic glioma patients explored and confirmed) or (2) a covariate threshold (eg, age cutoff value of 65, 70, and 75 years old for supramaximal explored, selected, and confirmed).

A third advantage of adaptive designs is subgroup enrichment.^73,74^ This means the subgroup of patients most likely to benefit from the treatment is selected. Although this is often a 2-step approach in which the first study post-hoc identifies this subgroup, and a second study is prospectively “enriched” for this subgroup, these 2 steps can be combined into 1 study with an adaptive design. This allows individual patients quicker access to the most effective treatment and decreases unnecessary exposure to less effective treatments. Potential downsides of adaptive designs are the higher costs due to added support requirements from data managers and statistics staff, and the risk of operation bias because caregivers and surgeons might learn which treatment is better during the trial.

### (2) Prospective, Exploratory Subgroup Analyses (Consistency Assessment)

The second type of subgroup analysis is the consistency assessment. Its primary aim is to examine if a treatment benefit demonstrated in an RCT, can be extrapolated to one or multiple pre-defined subgroups (treatment effect homogeneity). For instance, when a new surgical strategy is implemented and its effects are measured in the overall cohort, it could be useful to evaluate if the effects are the same in all the relevant subgroups. The RCT by Stummer et al. on using 5-aminolevulinic acid (5-ALA) in glioblastoma surgery is an example of this type of analysis.^3^ They found that the 6-month PFS was higher in the 5-ALA arm than in the conventional surgery arm (41.0% vs. 21.1%, *P* = .003). After stratifying these analyses for the pre-defined subgroups based on age, they found that this beneficial effect was consistent for younger and older patients (≤55 vs. < 55 years). To make the consistency assessment results easily interpretable, making a forest plot can be helpful. A forest plot summarizes the treatment effects across different subgroups in one comprehensive figure. This makes the results across the subgroups easier to digest for the reader and allows for examination if indeed there is treatment effect homogeneity.^62^ However, because these results can be underpowered, they must be interpreted cautiously.

### (3) Post-hoc and Exploratory Subgroup Analyses

The third type of subgroup analysis is the post hoc, exploratory analysis. Because it is sometimes impossible to predefine all potentially important subgroups, some must be examined post-hoc (after completing the study). This can be true for electronic health records, claims databases, and registry studies. Importantly, even though the subgroups are identified post-hoc, the methods to identify them can be defined prospectively. Alternatively, relevant patient subgroups can be identified post-hoc with a systematic (disciplined) subgroup search.^61,75^ This search aims to find patient subgroups that have a stronger effect on the surgery or treatment than other patient subgroups. It is important to note that post-hoc analyses cannot be used for inferential (causal) conclusions: their findings always need to be validated in a subsequent prospective confirmatory study. Furthermore, they are prone to confounding and should only be used as hypothesis-generating analyses.

The GLIOMAP study is an example of a study that uses post-hoc, exploratory subgroup analyses, in this case, to stratify for baseline prognostic imbalance. For this study, we compared the survival of awake craniotomy versus asleep resection using Kaplan-Meier curves.^14^ For the overall cohort and some of the matched subgroups, we observed “crossing curves.” This simply means that the Kaplan-Meier curves crossed each other and can indicate non-proportional hazards. One way to address this issue is to stratify the analysis for an important prognostic variable, molecular factors (MGMT methylation status and IDH mutation status). After stratifying the analyses for these molecular factors, the survival curves separated without crossover. Other reasons to use post-hoc analyses are to investigate if certain subgroups may benefit from the intervention (when the overall effect was negative), those that may benefit the most from the intervention (when the overall effect was positive), or have a different safety profile.

Therefore, the primary aim of post-hoc exploratory subgroup analyses is to investigate these differences between subgroups (treatment effect heterogeneity) and to generate new hypotheses.

In statistical terms, treatment effect heterogeneity is defined as interaction: the treatment effect differs between subgroups because there is an interaction between the treatment and the subgroup covariate.^76^ The GLIOMAP study might illustrate this.^14^ One of the study’s aims was to evaluate if awake craniotomy was predictive of complete resection of the contrast-enhancing tumor. We used multiple multivariable logistic regression analyses in the overall cohort and subgroups for age, preoperative NIHSS score, and preoperative KPS. The regression analyses showed that awake craniotomy was predictive of complete resection in the overall cohort (odds ratio [OR] 1.88, *P* = .013), and the subgroups aged <70 (OR 1.86, *P* = .028), aged ≥70 (OR 2.31, *P* = .012), NIHSS 0–1 (OR 1.97, *P* = .038), and KPS 90–100 (OR 2.44, *P* = .0080). However, no significant effect was found in the subgroups of NIHSS ≥ 2 (OR 1.35, *P* = .66), and KPS ≤ 80 (OR 2.19, *P* = .18). We then used an interaction term between the treatment (awake craniotomy) and the subgroup covariate (age, NIHSS, or KPS) to study treatment effect heterogeneity among the subgroups. The interaction term is coded as a “*” between the treatment and subgroup covariate. The null hypothesis of the interaction is that test the treatment effect is the same for different covariate values, for example, younger or older patients. In our analysis, the interaction terms were nonsignificant for awake*age (*P* = .77), awake*NIHSS (*P* = .50), and awake*KPS (*P* = .47). This indicates no statistical difference in the treatment effect of awake craniotomy on complete resection across these subgroups. In other words, the association between awake craniotomy and complete resection is similar for patients irrespective of age, preoperative NIHSS score, or preoperative KPS. The fact that we observed a nonsignificant effect in 2 subgroups (NIHSS ≥ 2 and KPS ≤ 80) suggests that the analyses for these subgroups might have been underpowered. This may have led to a type II error (false negative finding due to insufficient power). A common mistake is to claim that there is treatment effect heterogeneity because the association (OR and hazard ratio) between the treatment and the outcome is different within each of the levels of the baseline variable. For example, testing the association between awake craniotomy and complete resection in younger patients and then separately in older patients does not answer if age influences this association. Only the interaction test (eg, awake*age) determines if the effect in younger patients is different than in older patients. The theoretical reasons for this have been explained in previous papers.^30,77^

Interpreting post-hoc subgroup analyses can be a challenge. It is often helpful to evaluate if certain factors have been pre-specified. This provides insight and may benefit the credibility of the subgroup findings. Examples of factors that can be pre-specified are the rationale for the subgroup analysis (such as clinical or biological plausibility), the effect size and direction, covariate levels, and cutoff values, the statistical methods that will be used, or the endpoint that will be studied. It is important to keep in mind that plausibility is not always reliable: most subgroups are comprised of well-known predictive or prognostic factors, and it is therefore very common for these subgroups to be considered “plausible.” One way to address this is to prospectively discuss a range of factors that would be by their underlying mechanism the most probable factors, along with their direction of effect.

## Multiplicity Correction

Post-hoc analyses are often done with several subgroups. In an extreme form, this can lead to “data dredging” or “fishing expeditions.” This means that multiple subgroups are analyzed with the deliberate goal of finding a subgroup that has a significant outcome. This is problematic because analyzing multiple subgroups (multiple testing) makes these analyses prone to false-positive findings (type I error). The risk of false-positive findings increases with the number of tests that are performed (read: the number of hypotheses that are tested, the number of subgroups that are studied).^62,64^ This can be explained by the fact that typically, each analysis has an α of 5% associated with it (the risk to find a false-positive finding). If 3 analyses are performed with an α of 5%, the overall risk of a false-positive finding increases to 15%. This underlines the fact that for a correct interpretation of post-hoc analyses, it is vital to know how many tests have been performed for the subgroup analyses. The elevated risk of false-positive findings due to multiple tests can be mitigated by correcting for doing multiple tests (multiplicity correction). For multiplicity correction, 3 methods can be used: lowering the significance level *α*, increasing the *p*-value, or widening the confidence interval of the individual tests. The goal of all 3 methods is to make it more difficult to reject the null hypothesis for an individual test (to get a significant result). This will decrease the risk of false-positive results. We will focus on the first method (lowering the significance level) because this is the most frequently used in daily practice. The approaches to do this are summarized in Figure 2B.

The most commonly used multiplicity correction method is the Bonferroni method. This method is relatively simple: it divides the significance level α for the individual tests by the total number of tests performed. For example, when 5 hypotheses are tested in a study with an overall significance level of 0.05, the Bonferroni method lowers the significance level for the individual tests to 0.01. This ensures that testing 5 hypotheses with a significance level of 0.01 or testing one hypothesis with a significance level of 0.05 will have the same probability of finding false-positive results. The Bonferroni method is a so-called single-step multiple-testing procedure. This means that with one single adjustment to the significance level, all the hypotheses of the study can be tested simultaneously (eg, all hypotheses are significant when *P* < .01, instead of *P* < .05). The limitation of this method is that it decreases the power of the individual tests significantly as the number of tests increases. In other words, it can make the significance level *α* too conservative (e,. 0.01 instead of 0.05 can be too strict statistically in some instances). Alternatives that carry greater power are stepwise procedures,^63,64,78^ which test the hypotheses in a particular order. This means that after each test, the significance level is adjusted based on the “data” of the first test, and with this new significance level, the next hypothesis is tested. Because the adjustment of the significance level of the test is based on the “data” of the previous test, these stepwise procedures are called data-driven. Examples of data-driven procedures are Holm’s method, Hochberg’s method, and Hommel’s method.^79–81^ The alternative to data-driven procedures is pre-specified procedures: the adjustment of the significance level is not based on the previous test but is pre-specified. Examples are the fixed-sequence procedure, the fallback procedure, and the chain procedure^63,64^ (Figure 2B)**.** The theory behind these methods falls outside of the scope of this paper.

These methods to correct for multiplicity (single-step or stepwise) are examples of sequential approaches. Their limitations are outlined in Figure 2B. An alternative to these methods is the alpha spending function, which offers a flexible approach by “spending” portions of the overall type I error rate (α, usually 0.05) across multiple tests over time, such as the O’Brien-Fleming and Pocock boundaries.^82–85^ This approach can be useful particularly when conducting interim analyses as part of an adaptive randomized trial. For example, rather than requiring a predetermined (fixed) plan for carrying out the interim analyses, it allows for flexibility in the timing and number of interim analyses. This makes it easier to deal with unplanned deviations in the trial or extra analyses without inflating the type I error. Furthermore, this approach can adapt to the trial data by adjusting the spending rate of α based on the results of previous analyses. For example, if an early analysis looks promising, a smaller portion of α can be spent early, leaving more room for later tests. This may result in a higher power (lower type II error caused by an underpowered analysis), especially for early or unexpected interim analyses, by avoiding the sometimes overly conservative nature of traditional sequential approaches (such as the Bonferroni).

## Application Examples of Subgroup Analyses in Neuro-Oncology

The choice for a certain method of subgroup analysis is dependent on the hypothesis that the researchers aim to test or the research question that they try to answer. This will influence the choice to confirm, explore, or discover specific subgroups. As described earlier, 2 common reasons to use subgroup analyses are to study the association between treatment and outcome in an already known subgroup (etiological analysis) or to discover important subgroups.

**Figure 3.**
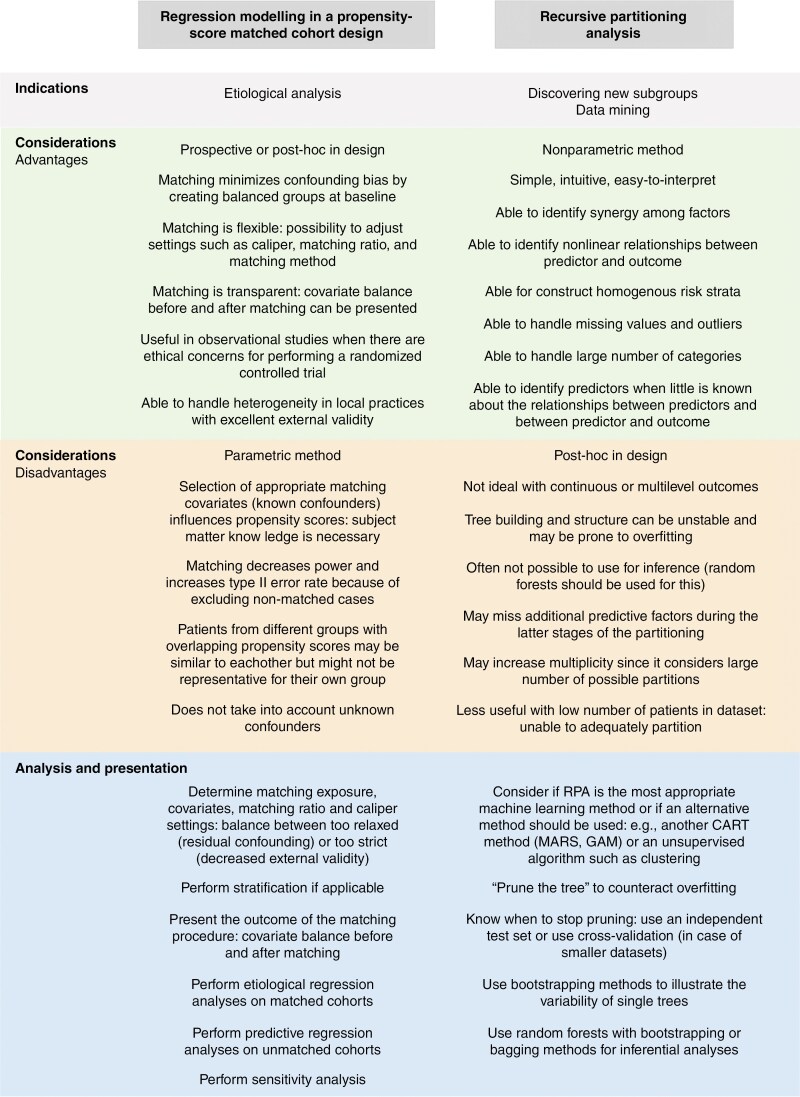
Pearls and pitfalls for 2 common subgroup analysis methods in surgical neuro-oncology.

**Figure 4.**
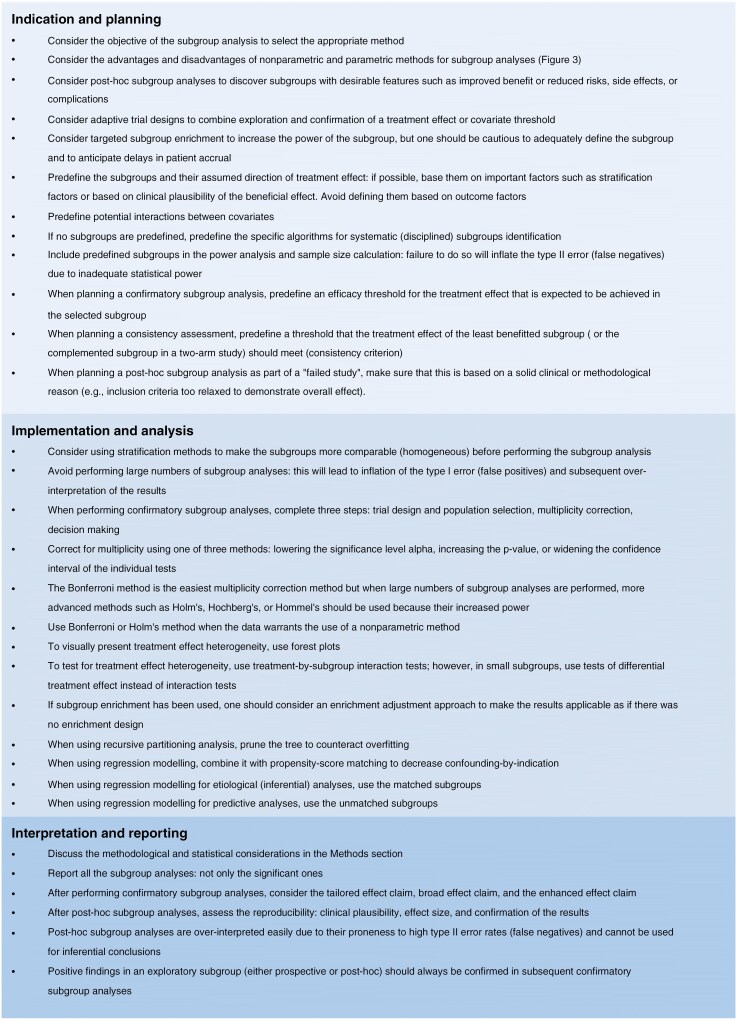
Recommendations for subgroup analyses in surgical neuro-oncology studies.

We will give 2 practical examples that illustrate these reasons along with the pearls and pitfalls on their indications, analysis, and interpretation. These are summarized in Figure 3. General recommendations for the planning, analysis, interpretation, and presentation of subgroup analyses are presented in Figure 4.

The study by Molinaro et al. published in 2020 illustrates the first reason.^16^ They investigated the value of minimizing the residual non-contrast enhancing (NCE) tumor volume in glioblastoma patients. The objective of this study was to assess the association of minimizing residual volume and survival while considering molecular and clinical factors. To this end, a variation of recursive partitioning analysis (RPA, a form of classification and regression trees [CART]) called partDSA was used to discover 4 distinct patient subgroups based on molecular and clinical data.^16,86^ Each of these subgroups corresponded to different survival outcomes. These CART techniques are often displayed as the branches of a tree because they split the data into several smaller patient subgroups, hence the name “classification tree.” The tree starts with a root node at the top of the tree. This root node includes all the available training data. From this root node, a split in the tree occurs and is based on the variable which increases the homogeneity of outcome (here, survival) in the resulting 2 new nodes (in this case: temozolomide after surgery: yes or no). These nodes can either be terminal or nonterminal nodes. Terminal nodes are nodes after which that specific branch of the tree stops: there are no more splits after this node. These nodes represent a distinct subgroup in the data. In this study, the first terminal node occurred after the first split: patients who had not undergone temozolomide after surgery formed a separate subgroup. Nonterminal nodes are nodes after which the branching of the tree continues. In this practical example, patients who had undergone temozolomide after surgery were split again for IDH status, age at diagnosis, and residual NCE tumor after surgery. In the end, all the cases included in the learning set end up at one specific terminal node and the partition—the set of all terminal nodes—is completed. In the Molinaro study,^16^ this resulted in 4 distinct subgroups of patients, based on 4 different nonterminal nodes: patients who did not receive temozolomide after surgery (subgroup 1), patients who received temozolomide, had an IDH wildtype tumor, and were older than 65 years old (subgroup 2), patients who received temozolomide, had an IDH wildtype tumor, were 65 years or younger, and had a residual NCE tumor of more than 5.4 ml (subgroup 3), and patients who received temozolomide and had an IDH mutated (IDHmt) tumor or those who received temozolomide, had an IDH wildtype (IDHwt) tumor, were 65 years or younger, and had a residual NCE tumor of less than 5.4 ml (subgroup 4). For this latter subgroup, the overall survival was similar between the subset of IDHwt tumors and the IDHmt tumors. Note that the partDSA algorithm differs from CART in that it inherently combines subgroups to maximize the homogeneity within a terminal node, rather than separate subgroups with similar outcomes. This is illustrated in our example study by the fact that subgroup 4 was formed by 2 distinct types of patients: IDHmt patients, and younger IDHwt patients with supramaximal resection.

There are a few challenges with RPA.^87^ The first is the risk of overfitting, that is fitting the data not only on the actual signal within the data but also on the noise. If the data are “overfit”: they only fit on the training data and not on other data, such as testing data or validation data (often because the model captures noise rather than the underlying pattern, which may lead to poor generalizability). To counteract overfitting, it is best not to have too many branches (subgroups). Therefore, like a tree, it can be pruned: finding a subtree of the “first draft” of the tree that is the best at predicting the outcome and is relatively protected to overfitting. However, the important part is deciding when to stop. There are 2 ways to test this: using an independent test set, or cross-validation (preferred for smaller datasets). Both these tests work by testing different potential subtrees for their potential to reliably predict the subgroups in data other than the original training set that the tree was built on.

A random forest is a collection of individual trees which has excellent predictive abilities.^88^ As the name implies, this method works by building a large number of trees using random subsets of the data (eg, with bootstrapping or bagging methods^89–91^). The final prediction uses results from all the different trees (the individual trees should not be pruned like in RPA because otherwise, there will be a loss of information). Random forests are more stable than “single trees” as developed by RPA and are less susceptible to prediction errors. The choice between random forest versus “single-tree” RPA depends on a number of factors.^92^ One major advantage of single-tree RPA is the interpretability of the tree structure. Each branch represents a clear decision rule and the final leaves represent distinct subgroups of patients. This means that single-tree RPA is useful to identify a single set of subgroups, assuming that the structure of the subgroups is simple and interactions are limited. However, single-tree RPA is prone to overfitting the data, especially when the tree grows too large. Also, it uses a “greedy algorithm,” which means that it splits each node without considering the overall structure. This can lead to suboptimal subgroup identification if the most informative splits are not chosen early in the tree. Third, it can be unstable, meaning that small changes in the data can lead to significant changes in the tree structure.^93^ Random forests, however, can solve some of these problems of single-tree RPA. Multiple possible splits and interactions between variables, which handle complex nonlinear relationships and interactions between subgroups, “rank” the variables based on importance, and can visualize the relationship between variables. Because random forests combine the decisions and predictions of a large number of trees, they are more stable and less prone to overfitting, and their results are more generalizable than single trees.^94^ However, the downside of random forests is that they are more difficult to interpret than single trees (the overall model is a “black box”). Thus, the clear interpretability and identification of subgroups with single-tree RPA means that this method is ideal for clinical decision-making. Random forests, on the other hand, are particularly useful when evaluating which variables are most important in predicting the outcome (especially when there is inadequate clinical knowledge to select the variables).

The advantages and disadvantages of CART techniques are shown in Figure 3 along with their indications, organization, presentation, and interpretation. Other nonparametric methods exist to discover subgroups and have their own advantages and disadvantages. For example, some are better at handling continuous variables (multivariate adaptive regression splines [MARS]^95^), more useful when the relationship between the predictor variable and outcome is not linear (generalized additive models [GAM]^96^), or instead of these supervised machine learning algorithms, are unsupervised (clustering^97^).

The second common reason to use subgroup analyses is the etiological analysis to study the association between treatment and outcome. Examples of statistical methods that can be used for these analyses are linear regression models (for continuous outcomes), logistic regression models (for binary outcomes), and Cox proportional-hazards regression models (for censored outcomes). Rather than discovering subgroups with “data mining” methods such as CART, MARS, and GAM, subgroups that already have been defined by the researcher are analyzed.

It is vital that the homogeneity within and between subgroups is maximized before moving on to the actual data analysis. Homogeneity within the subgroup can be maximized by using stratification methods. In RCT, this is called stratified randomization.^98^ For example, Stummer et al. randomly assigned patients to the 5-ALA and control arm, while considering the stratification factors age (≤ 55 vs. > 55 years), KPS (70–80 vs. > 80), eloquence (non-eloquent vs. eloquent), and study surgeon.^3^ This means that the randomization method will make sure that the patients in both trial arms are evenly balanced for these factors. Observational studies can mimic the stratification randomization design by first stratifying patients based on pre-defined factors (eg, age and functional status) before performing the analysis. An example can be observed in the GLIOMAP study and its supplementary analysis in which we stratified patients for age, preoperative neurological status, and preoperative functional status before moving on to the actual analysis.^14,19^

Homogeneity between subgroups can be improved by using matching methods. The most commonly used method is propensity score matching.^99^ This means that every patient gets assigned a propensity score. In the GLIOMAP study, this propensity score corresponded to the probability of having a certain exposure (awake craniotomy) based on their individual set of covariates (eg, age, functional status, etc).^14^ Patients from the group with the exposure (awake craniotomy) were matched with the patients from the group without the exposure (asleep resection) based on their propensity score (their individual set of covariates). This method acts as a countermeasure for confounding bias, a bias caused by the prognostic imbalance in the patient’s covariates (eg, if awake brain surgery is found to be associated with longer survival, but in particular younger patients undergoing awake brain surgery: it is now unknown if the longer survival should be attributed to the intervention [awake brain surgery] or the confounding covariate [age]). It is good practice to show in appendix tables the outcome of the matching procedure, that is the propensity scores for the unmatched and matched cohorts. It should be noted that propensity score matching has 2 major drawbacks: it only addresses known confounders, and it affects the statistical power because the available sample size is decreased after matching.

After maximizing homogeneity within and between subgroups, the second step is regression modeling. Regression models can be used for predictive or etiological analyses. Predictive analyses look for potential predictors in the dataset, for example, which baseline factors are predictive for a certain postoperative outcome. These analyses should be performed on unmatched cohorts to allow for optimum identification of the predictors in the “raw” data. Etiological analyses (also called inferential analyses) aim to determine causal relationships between covariates and outcomes, for example, which baseline factors are associated with a certain postoperative outcome. Therefore, these analyses should be performed on the matched cohorts to adequately counteract confounding bias. Further adjustment might be needed if certain variables were unstable in the matching procedure: these can be included in the subsequent regression to adjust for them adequately (sensitivity analysis). For example, in the GLIOMAP study, the molecular factors (MGMT methylation status, IDH mutation status) proved to be unstable in the matching procedure due to missing data.^14^ We therefore included these factors in the regression analyses as a sensitivity analysis to adjust for them. It is considered good practice to present the regression analyses without and without these factors for a reliable interpretation of the results.

## Conclusions

The surgical and nonsurgical treatment options for neuro-oncological patients are rapidly expanding. The neuro-oncological community is working together on a number of promising studies to try to improve the care for individual brain tumor patients. In this current era of personalized neuro-oncology, subgroup analyses are necessary to determine the optimum surgical treatment strategy for individual patients, especially given the fact that truly randomized studies, even unblinded, are challenging to conduct when dealing with surgical questions. However, subgroup analyses must be used with consideration and care because they have important potential risks. We aimed to give the reader for the first time a complete overview of the practical and statistical considerations regarding these analyses in neuro-oncology studies. We examined the pearls and pitfalls and evaluated the special considerations that are unique to surgical neuro-oncological studies. This paper can be used for other medical fields as well, even though the examples given were pertinent to neuro-oncology. The goal of this comprehensive guide was to assist with practical examples of how to design, use, and interpret subgroup analyses.
